# Supplementary material for: Endosome-associated Rab GTPases control distinct aspects of neural circuit assembly
Source: eLife. 2026 Oct 13;14:RP109394. doi: 10.7554/eLife.109394 (PMC13661049; doi:10.7554/eLife.109394)
Supplement: Supplementary file 2 [file elife-109394-supp1.docx]

| Figure | Genotype |
| --- | --- |
| Figure 1F–J | **Control**: *UAS-lacZ /+*; *VT033006-GAL4, GMR86C10-LexA, LexAop-mtdTomato, Or98a-mCD8-GFP, Or92a-CD2/+*  **Rab5 DN #1**: *UAS-Rab5.S43N* (BDSC 42703) */+*; *VT033006-GAL4, GMR86C10-LexA>LexAop-mtdTomato, Or98a-mCD8-GFP, Or92a-CD2/+*  **Rab5 DN #2**: *VT033006-GAL4, GMR86C10-LexA, LexAop-mtdTomato, Or98a-mCD8-GFP, Or92a-CD2/UAS-Rab5.S43N* (BDSC 42704)  **Rab11 DN #1**: *VT033006-GAL4, GMR86C10-LexA, LexAop-mtdTomato, Or98a-mCD8-GFP, Or92a-CD2/UASp-YFP.Rab11.S25N* (BDSC 23261)  **Rab11 DN #2**: *UASp-YFP.Rab11.S25N* (BDSC 9792) */+*; *VT033006-GAL4, GMR86C10-LexA, LexAop-mtdTomato, Or98a-mCD8-GFP, Or92a-CD2/+*  **Rab7 DN**: *VT033006-GAL4, GMR86C10-LexA>LexAop-mtdTomato, Or98a-mCD8-GFP, Or92a-CD2/UASp-YFP.Rab7.T22N* (BDSC 9778) |
| Figure 1-figure supplement 3A–E | **Control**: *UAS-lacZ /+*; *VT033006-GAL4, GMR86C10-LexA, LexAop-mtdTomato, Or98a-mCD8-GFP, Or92a-CD2/+*  **Rab4 DN:** *UASp-YFP.Rab4.S22N* (BDSC 9768)*/+*; *VT033006-GAL4, GMR86C10-LexA, LexAop-mtdTomato, Or98a-mCD8-GFP, Or92a-CD2/+*  **Rab21 DN:** *UAST-YFP.Rab21.T27N* (BDSC 23241)*/+*; *VT033006-GAL4, GMR86C10-LexA, LexAop-mtdTomato, Or98a-mCD8-GFP, Or92a-CD2/+*  **Rab35 DN:** *UAST-YFP.Rab35.S22N* (BDSC 9819)*/+*; *VT033006-GAL4, GMR86C10-LexA, LexAop-mtdTomato, Or98a-mCD8-GFP, Or92a-CD2/+* |
| Figure 2, Figure 3,  Figure 4 | **Control**: *UAS-mCD8-GFP, hsFlp122/+* (or *Y*); *TubP-Gal80, FRT40A, GH146-GAL4, UAS-mCD8-GFP/FRT40A*  ***Rab5^—/—^***: *UAS-mCD8-GFP, hsFlp122/+* (or *Y*); *TubP-Gal80, FRT40A, GH146-GAL4, UAS-mCD8-GFP/ Rab5^2^, FRT40A* |
| Figure 5 | *UAS-mCD8-GFP, hsFlp122/+* (or *Y*); *TubP-Gal80, FRT40A, GH146-GAL4, UAS-mCD8-GFP/FRT40A; UAS-2xFYVE-mCherry/+* |
| Figure 6 and Figure 6-figure supplement 1 | **Control**: *hsFlp122, QUAS-mCD8-GFP/+* (or *Y*);; *GH146-QF, FRT82B, TubP-QS/FRT82B*  ***Rab7^—/—^***: *hsFlp122, QUAS-mCD8-GFP/+* (or *Y*);; *GH146-QF, FRT82B, TubP-QS/FRT82B, Rab7^GAL4-KO^*  ***Rab7^—/—^+Rab7***: *hsFlp122, QUAS-mCD8-GFP/+* (or *Y*); *QUAS-mCherry-Rab7/+*; *GH146-QF, FRT82B, TubP-QS/FRT82B, Rab7^GAL4-KO^*  **Rab7 OE:** *UAS-mCD8-GFP, hsFlp122/+* (or *Y*); *TubP-Gal80, FRT40A, GH146-GAL4, UAS-mCD8-GFP/FRT40A; UAS-mCherry-Rab7/+* |
| Figure 6-figure supplement 2 | **Control**: *GH146-FLP/UAS-FRT10-stop-FRT10-3xHalo7-CAAX; 71B05-GAL4/UAS-lacZ*  **Rab7-DN**: *GH146-FLP/UAS-FRT10-stop-FRT10-3xHalo7-CAAX; 71B05-GAL4/UASp-YFP.Rab7.T22N* (BDSC 9778) |
| Figure 7, Figure 7-figure supplement 1A, B,  Figure 8,  Figure 9,  Figure 10 | **Control**: *UAS-mCD8-GFP, hsFlp122*; *GH146-GAL4/+*; *FRT82B, TubP-Gal80/FRT82B*  ***Rab11^—/—^***: *UAS-mCD8-GFP, hsFlp122*; *GH146-GAL4/+*; *FRT82B, TubP-Gal80/FRT82B, Rab11^EP3017^*  ***Rab11^—/—^+ Rab11***: *UAS-mCD8-GFP, hsFlp122*; *GH146-GAL4/UAS-mCherry-Rab11*; *FRT82B, TubP-Gal80/FRT82B, Rab11^EP3017^* |
| Figure 7-figure supplement 1C-G | **Control**: *UAS-mCD8-GFP, hsFlp122*; *MZ19-GAL4, UAS-mCD8-GFP/+*; *FRT82B, TubP-Gal80/FRT82B*  ***Rab11^—/—^***: *UAS-mCD8-GFP, hsFlp122*; *MZ19-GAL4, UAS-mCD8-GFP/+*; *FRT82B, TubP-Gal80/FRT82B, Rab11^EP3017^* |

**Supplementary File 1. Complete list of genotypes used in this study**
