# Supplementary material for: Endosome-associated Rab GTPases control distinct aspects of neural circuit assembly
Source: eLife. 2026 Oct 13;14:RP109394. doi: 10.7554/eLife.109394 (PMC13661049; doi:10.7554/eLife.109394)
Supplement: Supplementary file 3 [file elife-109394-supp2.docx]

| glomerulus | | all groups | control vs *Rab7* *^–/–^* | control vs  *Rab7* *^–/–^+Rab7* | *Rab7* *^–/–^* vs  *Rab7* *^–/–^+Rab7* |
| --- | --- | --- | --- | --- | --- |
| DL1 | p-value | 0.0264 | 0.279 | 1 | 0.279 |
|  | significance | * | n.s. | n.s. | n.s. |
| DA3 | p-value | 1 | 1 | 1 | 1 |
|  | significance | n.s. | n.s. | n.s. | n.s. |
| DC2 | p-value | 0.00372 | 0.0221 | 0.279 | 1 |
|  | significance | ** | * | n.s. | n.s. |
| D | p-value | 1 | 1 | 1 | 1 |
|  | significance | n.s. | n.s. | n.s. | n.s. |
| VA3 | p-value | 1 | 1 | 1 | 1 |
|  | significance | n.s. | n.s. | n.s. | n.s. |
| DC3 | p-value | 1 | 1 | 1 | 1 |
|  | significance | n.s. | n.s. | n.s. | n.s. |
| VA1d | p-value | 1 | 1 | 1 | 1 |
|  | significance | n.s. | n.s. | n.s. | n.s. |
| VM2 | p-value | 1 | 1 | 1 | 1 |
|  | significance | n.s. | n.s. | n.s. | n.s. |
| DM6 | p-value | 0.0244 | 0.145 | 1 | 0.581 |
|  | significance | * | n.s. | n.s. | n.s. |
| VA1v | p-value | 1 | 1 | 1 | 1 |
|  | significance | n.s. | n.s. | n.s. | n.s. |
| DL2d | p-value | 0.661 | 1 | 1 | 1 |
|  | significance | n.s. | n.s. | n.s. | n.s. |
| DL2v | p-value | 1 | 1 | 1 | 1 |
|  | significance | n.s. | n.s. | n.s. | n.s. |
| DA4lm | p-value | 1 | 1 | 1 | 1 |
|  | significance | n.s. | n.s. | n.s. | n.s. |
| DA4l | p-value | 1 | 1 | 1 | 1 |
|  | significance | n.s. | n.s. | n.s. | n.s. |
| DA2 | p-value | 1 | 1 | 1 | 1 |
|  | significance | n.s. | n.s. | n.s. | n.s. |
| VA6 | p-value | 1 | 1 | 1 | 1 |
|  | significance | n.s. | n.s. | n.s. | n.s. |
| DM5 | p-value | 1 | 1 | 1 | 1 |
|  | significance | n.s. | n.s. | n.s. | n.s. |
| VA2 | p-value | 1 | 1 | 1 | 1 |
|  | significance | n.s. | n.s. | n.s. | n.s. |
| DC1 | p-value | 1 | 1 | 1 | 1 |
|  | significance | n.s. | n.s. | n.s. | n.s. |
| VC2 | p-value | 1 | 1 | 1 | 1 |
|  | significance | n.s. | n.s. | n.s. | n.s. |
| VA4 | p-value | 1 | 1 | 1 | 1 |
|  | significance | n.s. | n.s. | n.s. | n.s. |
| DL5 | p-value | 0.443 | 1 | 1 | 1 |
|  | significance | n.s. | n.s. | n.s. | n.s. |
| VC4 | p-value | 1 | 1 | 1 | 1 |
|  | significance | n.s. | n.s. | n.s. | n.s. |
| VM4 | p-value | 1 | 1 | 1 | 1 |
|  | significance | n.s. | n.s. | n.s. | n.s. |
| VL2a | p-value | 1 | 1 | 1 | 1 |
|  | significance | n.s. | n.s. | n.s. | n.s. |

**Supplementary File 2. Complete statistical analysis for *Rab7* mutant adPN neuroblast clones, related to Figure 6—figure supplement 1.**

Statistical significance was assessed using the Fisher-Freeman-Halton test for overall group comparisons, followed by pairwise Fisher's exact tests with Benjamini-Hochberg correction for multiple comparisons.
