# Supplementary material for: Endosome-associated Rab GTPases control distinct aspects of neural circuit assembly
Source: eLife. 2026 Oct 13;14:RP109394. doi: 10.7554/eLife.109394 (PMC13661049; doi:10.7554/eLife.109394)
Supplement: Supplementary file 4 [file elife-109394-supp3.docx]

| glomerulus | | all groups | control vs *Rab11^–/–^* | control vs  *Rab11^–/–^ +Rab11* | *Rab11^–/–^* vs  *Rab11^–/–^ +Rab11* |
| --- | --- | --- | --- | --- | --- |
| DL1 | p-value | 8.27E-05 | 0.005 | 1 | 0.00648 |
|  | significance | *** | ** | n.s. | ** |
| DA3 | p-value | 0.000271 | 0.005 | 0.937 | 0.0309 |
|  | significance | *** | ** | n.s. | * |
| DC2 | p-value | 0.43 | 0.937 | 1 | 0.937 |
|  | significance | n.s. | n.s. | n.s. | n.s. |
| D | p-value | 0.00149 | 0.0233 | 1 | 0.0233 |
|  | significance | ** | * | n.s. | * |
| VA3 | p-value | 0.00149 | 0.0233 | 1 | 0.0233 |
|  | significance | ** | * | n.s. | * |
| DC3 | p-value | 0.171 | 0.626 | 1 | 0.666 |
|  | significance | n.s. | n.s. | n.s. | n.s. |
| VA1d | p-value | 8.27E-05 | 0.005 | 1 | 0.00648 |
|  | significance | *** | ** | n.s. | ** |
| VM7 | p-value | 0.088 | 0.289 | 1 | 0.342 |
|  | significance | n.s. | n.s. | n.s. | n.s. |
| VM2 | p-value | 8.27E-05 | 0.005 | 1 | 0.00648 |
|  | significance | *** | ** | n.s. | ** |
| DM6 | p-value | 0.43 | 0.937 | 1 | 0.937 |
|  | significance | n.s. | n.s. | n.s. | n.s. |
| VA1v | p-value | 8.27E-05 | 0.005 | 1 | 0.00648 |
|  | significance | *** | ** | n.s. | ** |
| DA1 | p-value | 0.0279 | 0.0598 | 1 | 0.131 |
|  | significance | * | n.s. | n.s. | n.s. |
| VC3 | p-value | 0.00173 | 1 | 0.0162 | 0.0233 |
|  | significance | ** | n.s. | * | * |
| DL2 | p-value | 0.00358 | 1 | 0.0598 | 0.0309 |
|  | significance | ** | n.s. | n.s. | * |
| DA2 | p-value | 0.171 | 0.196 | 1 | 0.937 |
|  | significance | n.s. | n.s. | n.s. | n.s. |
| VA6 | p-value | 0.724 | 0.937 | 1 | 1 |
|  | significance | n.s. | n.s. | n.s. | n.s. |
| VM5d | p-value | 1 | 1 | 1 | 1 |
|  | significance | n.s. | n.s. | n.s. | n.s. |
| VA7m | p-value | 0.43 | 1 | 0.937 | 0.937 |
|  | significance | n.s. | n.s. | n.s. | n.s. |
| DM5 | p-value | 0.43 | 1 | 0.937 | 0.937 |
|  | significance | n.s. | n.s. | n.s. | n.s. |
| DM3 | p-value | 1 | 1 | 1 | 1 |
|  | significance | n.s. | n.s. | n.s. | n.s. |
| DL5 | p-value | 1 | 1 | 1 | 1 |
|  | significance | n.s. | n.s. | n.s. | n.s. |
| VA4 | p-value | 1 | 1 | 1 | 1 |
|  | significance | n.s. | n.s. | n.s. | n.s. |
| VM4 | p-value | 0.724 | 0.937 | 1 | 1 |
|  | significance | n.s. | n.s. | n.s. | n.s. |
| VC4 | p-value | 0.43 | 0.626 | 0.937 | 1 |
|  | significance | n.s. | n.s. | n.s. | n.s. |
| DP1m | p-value | 1 | 1 | 1 | 1 |
|  | significance | n.s. | n.s. | n.s. | n.s. |

**Supplementary File 3. Complete statistical analysis for *Rab11* mutant adPN neuroblast clones, related to Figure 7 and Figure 7-figure supplement 1.**

Statistical significance was assessed using the Fisher-Freeman-Halton test for overall group comparisons, followed by pairwise Fisher's exact tests with Benjamini-Hochberg correction for multiple comparisons.
