# Supplementary material for: Endosome-associated Rab GTPases control distinct aspects of neural circuit assembly
Source: eLife. 2026 Oct 13;14:RP109394. doi: 10.7554/eLife.109394 (PMC13661049; doi:10.7554/eLife.109394)
Supplement: Peer review history

# Editor report: eLife Assessment

**DOI:** 10.7554/eLife.109394.3.sa0

**Authors:** P Robin Hiesinger

**Affiliations:** Institute for Biology Free University Berlin Germany

**Keywords:** Convincing, Valuable

This **valuable** paper by Dong et al. describes an analysis of mutant phenotypes of the Rab GTPases Rab5, Rab7 and Rab11 in *Drosophila* second order olfactory neuron development. The revised version presents a **convincing** characterization and comparison of the different Rab mutants on projection neuron development, with clear differences for the three Rabs and by inference for the early, late and recycling endosomal functions executed by each.

# Referee report: Reviewer #1 (Public review)

**DOI:** 10.7554/eLife.109394.3.sa1

Summary:

Dong et al. present an in-depth analysis of mutant phenotypes of the Rab GTPases Rab5, Rab7, and Rab11 in *Drosophila* second-order olfactory neuron development. These three Rab GTPases are amongst the best-characterized Rab GTPases in eukaryotes and have been associated with major roles in early endosomes, late endosomes, and recycling endosomes, respectively. All three have been investigated in *Drosophila* neurons before; however, this study provides the most detailed characterization and comparison of mutant phenotypes for axonal and dendritic development of fly projection neurons to date. In addition, the authors provide excellent high-resolution data on the distribution of each of the three Rabs in developmental analyses.

Strengths:

The strength of the work lies in the detailed characterization and comparison of the different Rab mutants on projection neuron development, with clear differences for the three Rabs and by inference for the early, late, and recycling endosomal functions executed by each.

Comments on revised version.

The authors conducted extensive revision experiments, especially to characterize developmental defects. Efforts to identify cargoes were not successful. The evidence is now convincing.

# Referee report: Reviewer #2 (Public review)

**DOI:** 10.7554/eLife.109394.3.sa2

Summary:

This study by Dong et al characterizes the roles of highly-expressed Rab GTPases Rab5, Rab7 and Rab11 in the development and wiring of olfactory projection neurons in *Drosophila*. This convincing descriptive study provides complementary approaches of Rab expression and localization profiling, conventional dominant negative mutants, and clonal loss of function mutants to address the roles of different endosomal trafficking pathways across circuit development. They show distinct distributions and phenotypes for different Rabs. Overall, the study sets the stage for future mechanistic studies in this well-defined central neuron.

Strengths:

Beautiful imaging in central neurons demonstrates differential roles of 3 key Rab proteins in neuronal morphogenesis as well as interesting patterns of subcellular endosome distribution. These descriptions will be critical for future mechanistic studies. The manuscript is well-written and explanatory, very accessible to a wide audience without sacrificing technical accuracy.

Comments on revised version.

The paper is greatly improved with new data and better-nuanced interpretation. The clarity of explanations for a non-*Drosophila* reader have been redone particularly well.

# Referee report: Reviewer #3 (Public review)

**DOI:** 10.7554/eLife.109394.3.sa3

Summary:

The authors aimed at a comprehensive phenotypic characterization of the roles of all Rab proteins expressed in PN neurons in developing *Drosophila* olfactory system. Important data are shown for a number of these Rabs with small/no phenotypes (in the Supplements) as well as the main endosomal Rabs, Rab5, 7, and 11 in the main figures.

Strengths:

The mosaic analysis is a great strength allowing visualization of small clones or single neuron morphologies. This also allows some assessment of cell autonomy of the observed phenotypes. The impact of the work lies in the comprehensiveness of the experiments. The rescue experiments are a strength. The added developmental data strengthen the impact of the paper.

Weaknesses:

The main weakness is that the experiments do not address the mechanisms that are affected by the loss of these Rab proteins, especially in terms of the most significant cargos. The insights thus do not extend far beyond what is already known from other work in many systems.

# Author comment: Author response

**DOI:** 10.7554/eLife.109394.3.sa4

**Authors:** Katherine X Dong, Yanbo Zhang, Hui Ji, David J Luginbuhl, Liqun Luo, Colleen N McLaughlin

**Affiliations:** Stanford University Stanford United States; Stanford University Stanford United States; Stanford University Stanford United States; Howard Hughes Medical Institute, Stanford University Stanford United States; Howard Hughes Medical Institute, Stanford University Stanford United States; Stanford University Stanford United States

The following is the authors’ response to the original reviews.

**Public Reviews:**

**Reviewer #1 (Public review):**

Summary:

Dong et al. present an in-depth analysis of mutant phenotypes of the Rab GTPases Rab5, Rab7, and Rab11 in *Drosophila* second-order olfactory neuron development. These three Rab GTPases are amongst the bestcharacterized Rab GTPases in eukaryotes and have been associated with major roles in early endosomes, late endosomes, and recycling endosomes, respectively. All three have been investigated in *Drosophila* neurons before; however, this study provides the most detailed characterization and comparison of mutant phenotypes for axonal and dendritic development of fly projection neurons to date. In addition, the authors provide excellent high-resolution data on the distribution of each of the three Rabs in developmental analyses.

Strengths:

The strength of the work lies in the detailed characterization and comparison of the different Rab mutants on projection neuron development, with clear differences for the three Rabs and by inference for the early, late, and recycling endosomal functions executed by each.

Weaknesses:

Some weakness derives from the fact that Rab5, Rab7, and Rab11 are, as acknowledged by the authors, somewhat pleiotropic, and their actual roles in projection neuron development are not addressed beyond the characterization of (mostly adult) mutant phenotypes and developmental expression.

We would like to thank Reviewer #1 for their appreciation of our characterization of distinct Rab mutants.

**Reviewer #2 (Public review):**

Summary:

This study by Dong et al. characterizes the roles of highly-expressed Rab GTPases Rab5, Rab7, and Rab11 in the development and wiring of olfactory projection neurons in *Drosophila*. This convincing descriptive study provides complementary approaches to Rab expression and localization profiling, conventional dominantnegative mutants, and clonal loss-of-function mutants to address the roles of different endosomal trafficking pathways across circuit development. They show distinct distributions and phenotypes for different Rabs. Overall, the study sets the stage for future mechanistic studies in this well-defined central neuron.

Strengths:

Beautiful imaging in central neurons demonstrates differential roles of 3 key Rab proteins in neuronal morphogenesis, as well as interesting patterns of subcellular endosome distribution. These descriptions will be critical for future mechanistic studies. The cell biology is well-written and explanatory, very accessible to a wide audience without sacrificing technical accuracy.

Weaknesses:

The *Drosophila* manipulations require more explanation in the main text to reach a wide audience.

We appreciate Reviewer #2’s analysis of our work and thank them for their suggestions to improve the clarity of our manuscript.

**Reviewer #3 (Public review):**

Summary:

The authors aimed at a comprehensive phenotypic characterization of the roles of all Rab proteins expressed in PN neurons in the developing *Drosophila* olfactory system. Important data are shown for a number of these Rabs with small/no phenotypes (in the Supplements) as well as the main endosomal Rabs, Rab5, 7, and 11 in the main figures.

Strengths:

The mosaic analysis is a great strength, allowing visualization of small clones or single neuron morphologies. This also allows some assessment of the cell autonomy of the observed phenotypes. The impact of the work lies in the comprehensiveness of the experiments. The rescue experiments are a strength.

Weaknesses:

The main weakness is that the experiments do not address the mechanisms that are affected by the loss of these Rab proteins, especially in terms of the most significant cargos. The insights thus do not extend far beyond what is already known from other work in many systems.

We thank this reviewer for their feedback and appreciation of our genetic manipulations.

**Recommendations for the authors:**

**Reviewing Editor Comments:**

Consensus suggestions after discussion of all three reviewers:

All three reviewers agree that the morphological and phenotypic analysis of the fly olfactory neurons is a strength of the manuscript. The shared perceived weakness is that the experiments do not address the mechanisms that are affected by the loss of these Rab proteins, especially in terms of the most significant cargos; the findings are in line with a large body of literature.

The three reviewers feel that the manuscript could be strengthened greatly by adding data on an actual cargo (cell surface proteins?) and a more detailed analysis of the actual developmental origin (what happens when during axon and dendrite development) with respect to sorting of such cargo in the neurons they analyzed.

We appreciate the time and effort of all three of our reviewers and share their interest in both identifying Rab-regulated cargos as well as determining the developmental origins of the Rab phenotypes. We have added three additional main figures (new Figure 4, Figure 8, and Figure 9), two supplemental figures (Figure 1—figure supplement 1 and 2), two additional supplemental tables (Table S2 and S3), and five additional panels (in Figure 3 H–L) of mutant developmental phenotype analysis.

Regarding cargos, we also share the reviewers’ desire to identify cargos regulated by each Rab and made attempts to do so but were ultimately unable to achieve this goal. The main obstacles to this were: (1) it is not known which cell-surface proteins are most robustly endocytosed in PNs; without this knowledge it is difficult to identify candidates whose localization would predominantly reflect endosomal rather than plasma membrane distribution, making it challenging to detect changes in compartment-specific localization upon Rab perturbation; (2) reagents to evaluate cell-surface proteins in PNs are not cell-type-specific making it difficult to evaluate changes in their distribution in PNs; (3) tagged overexpressed proteins are either unavailable or expressed at levels too high to sensitively detect changes in their distribution. We have elaborated on each of these points below and feel that cargo identification, while an important future direction, is beyond the scope of the present study.

**Recommendations for the authors:**

**Reviewer #1 (Recommendations for the authors):**

There are a number of experiments and ideas that the authors might consider to further improve on this work.

(1) The idea, introduced by the authors in the introduction, that Rab-mediated recycling of cell surface proteins back to the membrane versus degradation is, of course, excellent and interesting. It is less clear how this applies to the present study. The functions of Rab5, Rab7, and Rab11 are so widespread, potentially affecting many signaling roles resulting in primary or secondary effects on membrane and even cytoskeletal regulation, that it remains unclear whether the mutant phenotypes are related to the recycling or degradation of cell surface receptors. To link the idea to experimentation, the authors have an excellent opportunity in their system to look at endogenously tagged cell surface proteins (or at least one example), many of which the Luo lab has characterized in these neurons, to minimally correlate cell surface protein defects to the observed developmental defects.

We understand this critique and share this reviewer’s interest in identifying the specific cargos regulated by each Rab during development. We attempted to use antibodies to evaluate changes in cell-surface protein localization in response to disrupting individual Rabs but were unable to reliably distinguish shifts in association with specific endosomal compartment as many available antibodies label cell-surface proteins expressed in antennal lobe cells beyond projection neurons (such as olfactory receptor neurons, glia, or local interneurons) which complicates analyses.

Additionally, although we have, in other work, generated multiple 'flp-on' tags for PN cell-surface proteins, these cannot be used in combination with the MARCM system, as it relies on a heat-shock-inducible flp to label singlePN clones. Heat shock would simultaneously induce tag expression in other cells expressing the tagged gene, preventing PN-type-specific detection. This incompatibility thus prevents us from simultaneously perturbing individual Rabs and tracking corresponding changes in surface-protein localization with single-cell resolution.

Moreover, for proteins that are not highly endocytosed, it is difficult to separate plasma-membrane from endosomal localization, and we currently do not know which cell-surface proteins are most robustly endocytosed in PNs. Thus, while we share the reviewer’s interest in identifying candidate cargos, technological limitations make it difficult to achieve this goal within the scope of the current study.

(2) The mutant phenotypes are mostly characterized based on adult outcomes. Maybe a little more can be learned about when and how Rab5, Rab7, or Rab11 function is locally required by characterizing the developmental processes that lead to, e.g., aberrant dendritic development in Rab5 and Rab11.

We also feel that charting the developmental origins of Rab mutant phenotypes is important. Prior to mid-pupal stage (around 48 hours after puparium formation), glomeruli in the antennal lobe have not yet assumed their stereotyped positions, which complicates analyses and interpretation; thus, many of our analyses are conducted at the adult stage. For *Rab11* mutants we did perform many developmental analyses to evaluate the origins of the axonal development (Figure 6—figure supplement 1) and dendrite elaboration phenotypes (Figure 5 J–L) we observed at the adult stage. We realize that the developing axonal analyses were in supplemental material where they could be missed. We have moved these data to the main figures (Figures 8 and 9) and emphasized these analyses. Further, we extended our *Rab5* mutant analyses to evaluate developmental phenotypes (Figure 3H– L and Figure 4). We believe that these new analyses have strengthened the manuscript.

(3) Regarding the subcellular localization analyses: a collection of endogenously tagged Rabs in *Drosophila* has been generated by Dunst et al. (2015), which is surprisingly not cited. Maybe the authors could consider looking at the endogenous localization of Rabs using the tagged version in parallel to their overexpressed tagged versions.

We have now cited and discussed this paper (line 82) and thank the reviewer for pointing out this omission. We previously attempted to evaluate these endogenously tagged Rab proteins in PNs; however, since PN dendrites project into the antennal lobe, a dense neuropil region containing PN dendrites, ORN axons, glial processes, and neurites of local interneurons, we are unable to resolve individual Rab puncta from cytosolic (non-vesicle associated Rabs) or evaluate Rab localization in a cell-type-specific manner. For this reason, we focused on evaluating the localization of tagged Rab proteins from UAS-transgenes using a MARCM rescue strategy. We directly addressed this in the text (starting on line 83).

(4) It is maybe not entirely surprising that Rab5 and Rab11 have the strongest phenotypes, as these have been implicated in early and recycling endosomal processes in basically all eukaryotic cells with major implications for signaling throughout development and function, often causing cell death (and in the case of Rab5, tumorigenic phenotypes in flies). By contrast, the *Drosophila* brain can develop in the absence of Rab7 (Cherry et al., 2013; also the reference for the Rab7 null mutant, not Chan et al., 2011). A key concern in any developing fly cell rendered mutant using clonal analysis is the perdurance of RNA or protein (ultimately even maternal contribution), which could be addressed by discussion or experimentally.

We thank the reviewer for pointing out our citation error, which we have now corrected.

However, we note that Cherry et al. (2013) found that loss of Rab7 causes pupal lethality at stages prior to completion of 50–80% of development and can also cause embryonic lethality when maternal Rab7 contribution is blocked. This indicates that the whole organism cannot fully develop in the absence of Rab7. And while Cherry and colleagues did evaluate overall brain morphology in *Rab7* mutant pupae, they did not look at the development of individual cell types. So, it is still unclear how loss of this GTPase affects the development of individual central nervous system neurons.

Thus, to understand whether Rab7 has functions in PN development, we used the QMARCM system to perform Rab7 LOF analyses in PN clones. While we did not observe any phenotypes in single-cell MARCM clones (Figure 6), we did see mild defects in neuroblast clones (Figure 6—figure supplement 1A–C). Since single-cell MARCM clones are more susceptible to RNA/protein perdurance, we further evaluated Rab7 function by expressing a Rab7 dominant-negative transgene in DL1-PNs using a DL1-specific GAL4 driver (Figure 6—figure supplement 2), which circumvents potential perdurance issues mentioned by this reviewer. Importantly, this same transgene produces dendrite targeting defects when expressed in all PNs (Figure 1I), confirming its efficacy. However, no phenotypes were observed when expression was restricted to DL1-PNs, suggesting that Rab7 may not be required in DL1-PNs for their dendrite targeting. Given that both *Rab7* mutant neuroblast clones and pan-PN expression of Rab7 dominant negative causes PN dendrite targeting defects we conclude that Rab7 is nonautonomously required for dendrite targeting of DL1-PNs.

We have softened our language with regards to the Rab7 analysis and have emphasized, and strengthened, our previous discussion of these points beginning on line 268 in the results section and on line 427 of the discussion.

**Reviewer #2 (Recommendations for the authors):**

(1) In Figure 1B, it would be useful to show the circuit over multiple developmental stages, rather than just in its final form.

We have added this to Figure 1; it is now panel C. Thank you for this suggestion.

(2) Expression analysis of Rabs in Figure 1C - how do these levels and ratios compare to the whole brain? Whole body?

Unfortunately, we are unable to evaluate how Rab expression in PNs compares to all other cells in the brain as there is no sequencing data available for this organ at this time point. We did compare the expression of endosomal Rabs between PNs and their presynaptic targets, ORNs. We found that many Rabs displayed similar expression patterns between these two cell types during development. We have added a new paragraph on this, beginning on line 100 and we added two additional supplemental figures (Figure 1—figure supplement 1 and 2).

(3) The authors should include at least a few sentences comparing the current approach and results to previous comprehensive Rab protein expression analysis, for example, in PMID 17409086, 22000105, 22844416, and 33666175.

Thank you for pointing out this omission, we have amended it beginning on line 82.

(4) For the non-*Drosophila* reader (for example, a cell biologist working on endosomal traffic in cultured neurons), the paper is less accessible. Some examples:

We thank this reviewer for their suggestions for ways to clarify our work for the non-*Drosophila* reader. We have addressed each of their points.

(a) The severity difference between Rab5, Rab11, Rab7 and Rab4, Rab 21, Rab35 isn't immediately obvious from the images to someone who doesn't work with this system - does the brightness of the ectopic growths indicate the number of ectopically grown processes? It might help to have half a sentence to make this difference more accessible for the readers who aren't familiar with this system.

We have clarified this beginning on line 112.

(b) Figures 1D-E require more extensive description of the experimental setup with orthogonal expression systems than is provided briefly in the cartoon, figure legend, and supplement. For example, it should be noted what white vs blue represents in the marked glomeruli.

We have clarified this point beginning on line 114.

(c) There should be at least one sentence introducing what is marked and what it means when MARCM clones are first shown in Figure 2B, in addition to the supplemental figure.

We have added a detailed explanation of MARCM on line 155.

(d) It's not clear to a non-expert what the meaning is of no innervation of non-adPN glomeruli in wild-type in Figure 2E. This requires a sentence of explanation.

We have added additional details about this on line 159 and 169.

(e) Can a control image be shown for the experiment in 3B?

We have added an additional set of control images in Figure 3B on the left.

(5) The experiment measuring axonal projection to the lateral horn in Rab5 clones in Figure 3 J-L is underpowered (n=3 for mutant). While this may be due to the frequency of an overall projection defect as shown in Figure 3B, it makes it difficult to assess the robustness of the terminal phenotype. Further, for clarity, similar measurements (e.g., bouton width) should be aligned vertically between E-G and J-L.

We have performed additional analyses on Rab5 axons in the lateral horn and added them to a new Figure 4. The n’s are now n=10 for controls and n=7 for *Rab5* mutants. Additionally, we have aligned similar measurements in the figure panels and standardized the axes of each graph so that it is easier to compare between developmental stages.

(6) The argument that cell-type-specific phenotypes are due to distinct cargoes is weak. The same cargo could have different functions or signaling properties in different cell types (e.g., "Taken together, the distinct branching phenotypes observed in the mushroom body versus lateral horn suggest that Rab5 may regulate the trafficking of a distinct set of cargos in each axonal compartment"). Similarly, this argument is just one of many possibilities, as the effect could be quite indirect (eg via mis-regulated signal transduction): "Yet, the terminal boutons in Rab5 mutants were nearly 2-fold larger than those of controls (Figure 3G), suggesting Rab5 regulates the trafficking of cell-surface proteins that normally restrain bouton growth " and "Page 12 "Rab7mediated degradation does not have a major role in regulating axon or dendrite development" - should be softened since Rab7 may easily play an important but redundant role.

We have made all of these changes and removed references to trafficking of specific CSPs.

(7) Statistics need to be added to: Figure 3B, Figure 5D-E, Figure 2 - figure supplement 1C, Figure 4 - figure supplement 1E, F, Figure 5 - figure supplement 1A, E, Figure 6 - figure supplement 1K.

We thank this reviewer for pointing out this omission, we have added statistical measurements to our graphs.

As to not visually overwhelm readers with statistical measurements on already dense graphs (such as Figure 7B), we have added two supplemental tables (Table S2 and S3) that display all the results of all of the comparisons performed in the statistical tests.

We have cited this table in the figure legends and in-text figure references.

In addition to the methods, we have also added the exact statistical test and post-hoc tests (when applicable) to the figure legends.

(8) The BSDC identifier for the UAS-Rab11-mCherry stock may be incorrect.

It appears that some of the values in the ‘Identifiers’ column of the Key Resources table shifted downward. We have fixed this and appreciate that the reviewer pointed this out.
